# Practical Approach to Mixed Methods Research for Clinicians

**DOI:** 10.3126/nje.v9i1.24002

**Published:** 2019-03-31

**Authors:** Nishida Chandrasekharan

**Affiliations:** 1 Lecturer, Department of Orthopaedics, Government Medical College, Thrissur, Kerala.

As a clinician, finding the time as well as the opportunity to pursue and follow research actively is usually quite challenging. Moreover, with the passage of time, a focus on developing research falls by the wayside in our emphasis on patient care and the related demands on time and energy.

Over the last few decades, mixed methods research has increased in popularity as a third methodology, alongside pure qualitative and quantitative methods. Mixed methods research uses more than one research method, typically a combination of quantitative and qualitative methods [1].

Although mixed methods research has gained some traction in fields such as psychology and behavioral sciences, its application in other clinical fields (including trauma, orthopedics, or primary care) appears limited, possibly due to lack of awareness or inherent limitations in such studies. What are the potential pros and cons of MIXED METHODS RESEARCH, and is it a feasible approach to apply in current clinical scenarios?

Based on the principles of mixed methods research, it has been noted that on several levels, clinical practice does employ findings from both qualitative and quantitative research in a holistic manner, with a view to improving patient care and clinical skills. In a commentary by O’Cathain, the pragmatic approach of mixed methods research in answering complex questions that are encountered in health care has been found to be encouraging, with the number of mixed methods-based research studies increasing from 17% in the 1990s to around 30% in the 2000s [2].

In health systems, mixed methods research can help researchers and clinicians to view problems from multiple perspectives, develop a more complete understanding of a problem, “triangulate” results, and quantify hard-to-measure constructs. It also helps demonstrate contexts for trends, examine processes/experiences along with outcomes, and obtain a macro picture of a system [3].

Ozawa et al. believed that a mixed methods approach may be particularly helpful in low- and middle-income country settings to understand and improve health systems performance. They attribute this to various causes, including the presence of complex sociocultural factors with no clear frameworks or measurements, lack of qualitative data making it difficult to assess information from a qualitative study, logistic or financial obstacles to data collection, and a requirement for multiphasic studies [1].

Mixed methods research can help researchers, including clinicians, in developing survey tools, interventions, or programs ion the basis of qualitative study findings. Mixed methods research may be useful in identifying individuals eligible for further follow up or can describe the processes/reasons behind the quantitative results. Quantitative elements help understand the extent of a scenario and determine the representativeness of each finding, while qualitative studies illustrate stakeholder perspectives and provide a rationale for the performance of the system [4].

Nevertheless, mixed methods research is not without its disadvantages. Researchers have to be well versed in multiple methods and understand how to combine them effectively. A single researcher might find it difficult to conduct mixed methods research alone, especially if both methodologies are being used simultaneously. Some researchers question the validity of the analyses in mixed methods research since each methodology has a specific data set and analytical methods and believe that only pure qualitative or quantitative studies can be valid. Other issues include problems of “paradigm mixing” and financial/logistical issues [5].

Despite these limitations, mixed methods research holds considerable promise as a research methodology by compensating and enhancing the advantages and limitations of both qualitative and quantitative research. As it falls on the researcher to choose the study design, more encouragement and awareness via workshops and seminars regarding this methodology will be helpful to clinicians in the planning stage, and further debate and clarity is expected with establishment of further research.
